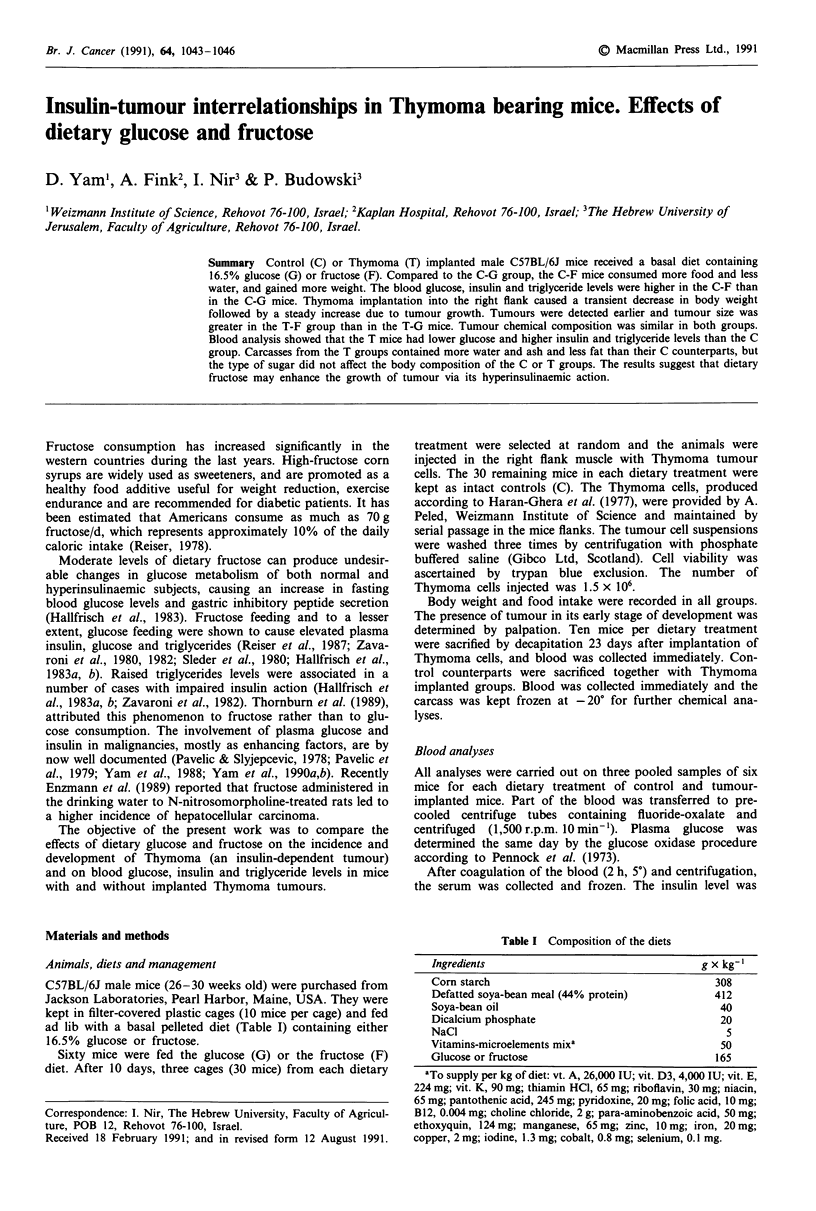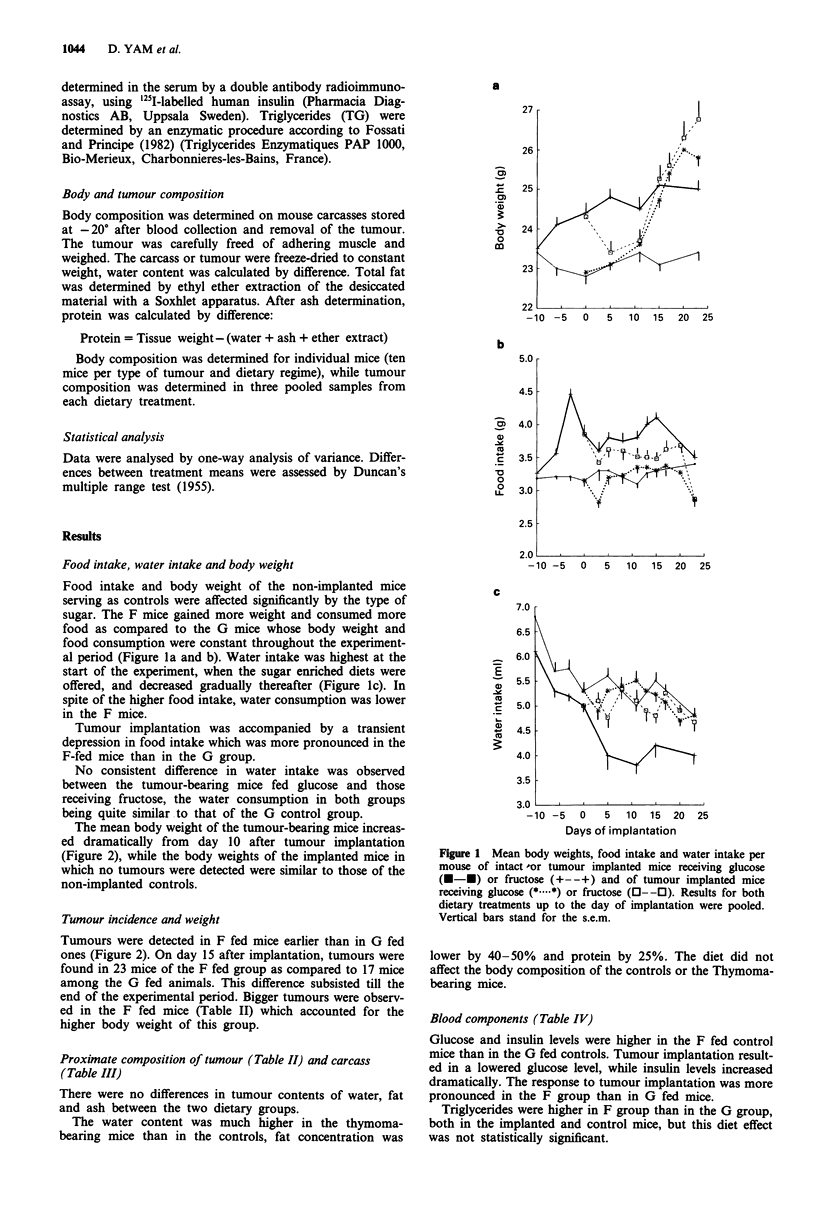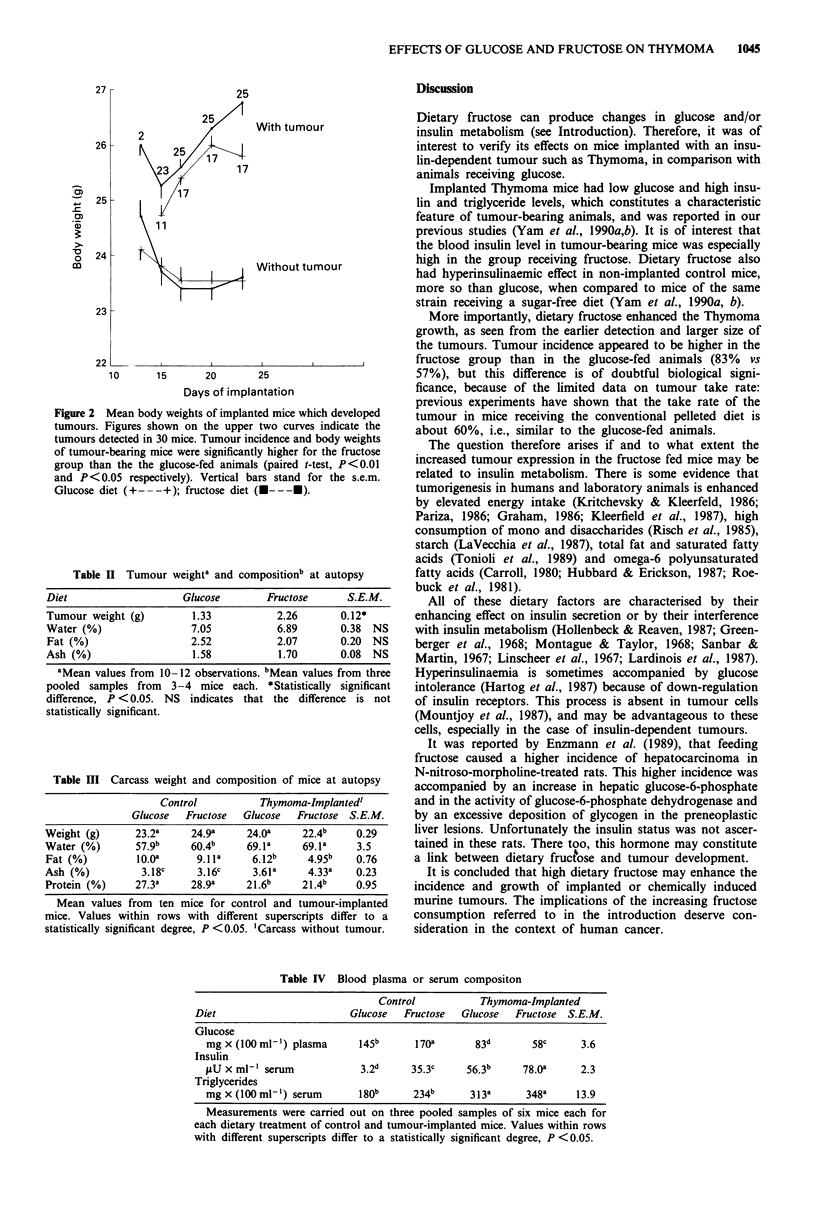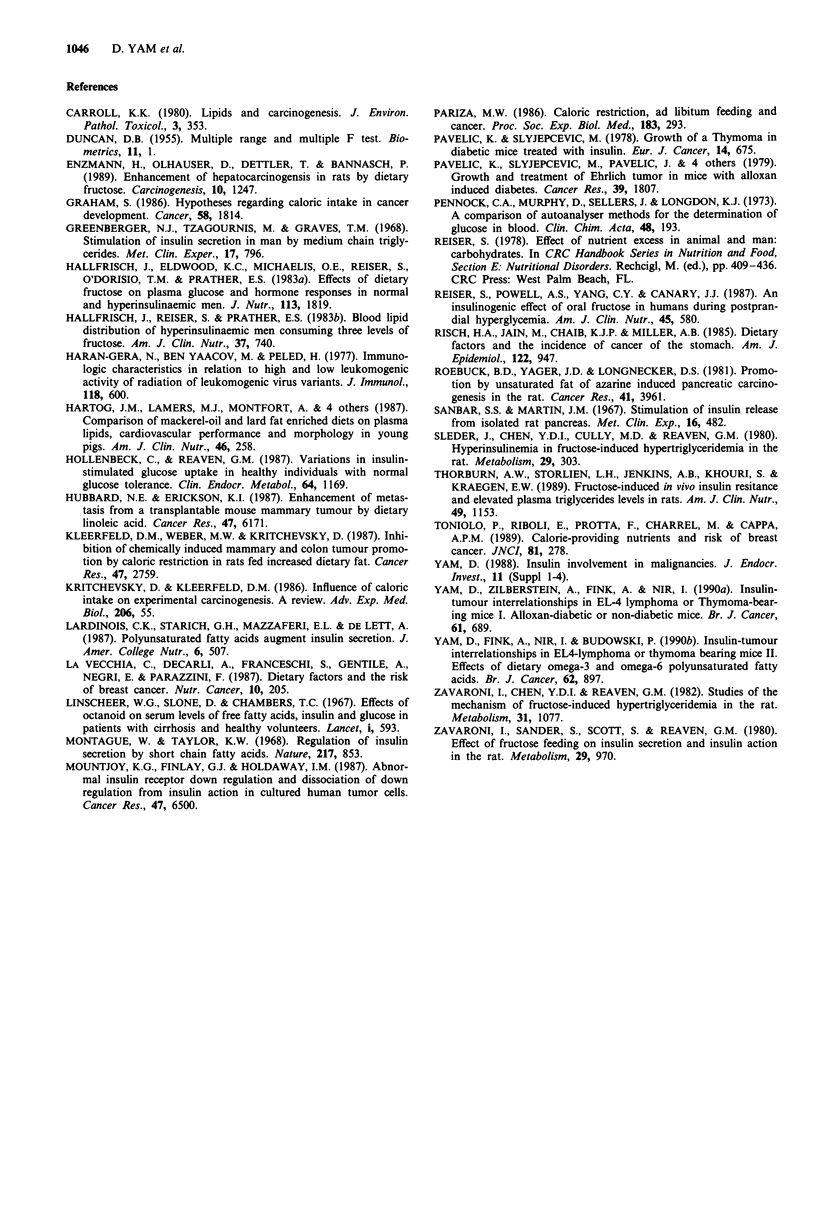# Insulin-tumour interrelationships in thymoma bearing mice. Effects of dietary glucose and fructose.

**DOI:** 10.1038/bjc.1991.462

**Published:** 1991-12

**Authors:** D. Yam, A. Fink, I. Nir, P. Budowski

**Affiliations:** Weizmann Institute of Science, Rehovot, Israel.

## Abstract

Control (C) or Thymoma (T) implanted male C57BL/6J mice received a basal diet containing 16.5% glucose (G) or fructose (F). Compared to the C-G group, the C-F mice consumed more food and less water, and gained more weight. The blood glucose, insulin and triglyceride levels were higher in the C-F than in the C-G mice. Thymoma implantation into the right flank caused a transient decrease in body weight followed by a steady increase due to tumour growth. Tumours were detected earlier and tumour size was greater in the T-F group than in the T-G mice. Tumour chemical composition was similar in both groups. Blood analysis showed that the T mice had lower glucose and higher insulin and triglyceride levels than the C group. Carcasses from the T groups contained more water and ash and less fat than their C counterparts, but the type of sugar did not affect the body composition of the C or T groups. The results suggest that dietary fructose may enhance the growth of tumour via its hyperinsulinaemic action.


*Br. J. Cancer* (1991), **64**, 1043–1046 © Macmillan Press Ltd., 1991

### Insulin-tumour interrelationships in Thymoma bearing mice. Effects of dietary glucose and fructose

D. Yam[1], A. Fink[2], I. Nir[3] & P. Budowski[3]

[1]*Weizmann Institute of Science, Rehovot 76-100, Israel;* [2]*Kaplan Hospital, Rehovot 76-100, Israel;* [3]*The Hebrew University of Jerusalem, Faculty of Agriculture, Rehovot 76-100, Israel.*

**Summary** Control (C) or Thymoma (T) implanted male C57BL/6J mice received a basal diet containing 16.5% glucose (G) or fructose (F). Compared to the C-G group, the C-F mice consumed more food and less water, and gained more weight. The blood glucose, insulin and triglyceride levels were higher in the C-F than in the C-G mice. Thymoma implantation into the right flank caused a transient decrease in body weight followed by a steady increase due to tumour growth. Tumours were detected earlier and tumour size was greater in the T-F group than in the T-G mice. Tumour chemical composition was similar in both groups. Blood analysis showed that the T mice had lower glucose and higher insulin and triglyceride levels than the C group. Carcasses from the T groups contained more water and ash and less fat than their C counterparts, but the type of sugar did not affect the body composition of the C or T groups. The results suggest that dietary fructose may enhance the growth of tumour via its hyperinsulinaemic action.

Fructose consumption has increased significantly in the western countries during the last years. High-fructose corn syrups are widely used as sweeteners, and are promoted as a healthy food additive useful for weight reduction, exercise endurance and are recommended for diabetic patients. It has been estimated that Americans consume as much as 70 g fructose/d, which represents approximately 10% of the daily caloric intake (Reiser, 1978).

Moderate levels of dietary fructose can produce undesirable changes in glucose metabolism of both normal and hyperinsulinaemic subjects, causing an increase in fasting blood glucose levels and gastric inhibitory peptide secretion (Hallfrisch *et al.*, 1983). Fructose feeding and to a lesser extent, glucose feeding were shown to cause elevated plasma insulin, glucose and triglycerides (Reiser *et al.*, 1987; Zavaroni *et al.*, 1980, 1982; Sleder *et al.*, 1980; Hallfrisch *et al.*, 1983*a*, *b*). Raised triglycerides levels were associated in a number of cases with impaired insulin action (Hallfrisch *et al.*, 1983*a*, *b*; Zavaroni *et al.*, 1982). Thornburn *et al.* (1989), attributed this phenomenon to fructose rather than to glucose consumption. The involvement of plasma glucose and insulin in malignancies, mostly as enhancing factors, are by now well documented (Pavelic & Slyjepcevic, 1978; Pavelic *et al.*, 1979; Yam *et al.*, 1988; Yam *et al.*, 1990*a*,*b*). Recently Enzmann *et al.* (1989) reported that fructose administered in the drinking water to N-nitrosomorpholine-treated rats led to a higher incidence of hepatocellular carcinoma.

The objective of the present work was to compare the effects of dietary glucose and fructose on the incidence and development of Thymoma (an insulin-dependent tumour) and on blood glucose, insulin and triglyceride levels in mice with and without implanted Thymoma tumours.

### Materials and methods

### *Animals, diets and management*

C57BL/6J male mice (26–30 weeks old) were purchased from Jackson Laboratories, Pearl Harbor, Maine, USA. They were kept in filter-covered plastic cages (10 mice per cage) and fed ad lib with a basal pelleted diet (Table I) containing either 16.5% glucose or fructose.

Sixty mice were fed the glucose (G) or the fructose (F) diet. After 10 days, three cages (30 mice) from each dietary treatment were selected at random and the animals were injected in the right flank muscle with Thymoma tumour cells. The 30 remaining mice in each dietary treatment were kept as intact controls (C). The Thymoma cells, produced according to Haran-Ghera *et al.* (1977), were provided by A. Peled, Weizmann Institute of Science and maintained by serial passage in the mice flanks. The tumour cell suspensions were washed three times by centrifugation with phosphate buffered saline (Gibco Ltd, Scotland). Cell viability was ascertained by trypan blue exclusion. The number of Thymoma cells injected was $1.5 \times 10^6$.

Body weight and food intake were recorded in all groups. The presence of tumour in its early stage of development was determined by palpation. Ten mice per dietary treatment were sacrified by decapitation 23 days after implantation of Thymoma cells, and blood was collected immediately. Control counterparts were sacrificed together with Thymoma implanted groups. Blood was collected immediately and the carcass was kept frozen at $-20°$ for further chemical analyses.

### *Blood analyses*

All analyses were carried out on three pooled samples of six mice for each dietary treatment of control and tumour-implanted mice. Part of the blood was transferred to pre-cooled centrifuge tubes containing fluoride-oxalate and centrifuged (1,500 r.p.m. 10 $min^{-1}$). Plasma glucose was determined the same day by the glucose oxidase procedure according to Pennock *et al.* (1973).

After coagulation of the blood (2 h, 5°) and centrifugation, the serum was collected and frozen. The insulin level was

**Table I** Composition of the diets

| *Ingredients* | *g* × $kg^{-1}$ |
|---|---|
| Corn starch | 308 |
| Defatted soya-bean meal (44% protein) | 412 |
| Soya-bean oil | 40 |
| Dicalcium phosphate | 20 |
| NaCl | 5 |
| Vitamins-microelements mix[a] | 50 |
| Glucose or fructose | 165 |

[a]To supply per kg of diet: vt. A, 26,000 IU; vit. D3, 4,000 IU; vit. E, 224 mg; vit. K, 90 mg; thiamin HCl, 65 mg; riboflavin, 30 mg; niacin, 65 mg; pantothenic acid, 245 mg; pyridoxine, 20 mg; folic acid, 10 mg; B12, 0.004 mg; choline chloride, 2 g; para-aminobenzoic acid, 50 mg; ethoxyquin, 124 mg; manganese, 65 mg; zinc, 10 mg; iron, 20 mg; copper, 2 mg; iodine, 1.3 mg; cobalt, 0.8 mg; selenium, 0.1 mg.

Correspondence: I. Nir, The Hebrew University, Faculty of Agriculture, POB 12, Rehovot 76-100, Israel.
Received 18 February 1991; and in revised form 12 August 1991.

determined in the serum by a double antibody radioimmunoassay, using $^{125}$I-labelled human insulin (Pharmacia Diagnostics AB, Uppsala Sweden). Triglycerides (TG) were determined by an enzymatic procedure according to Fossati and Principe (1982) (Triglycerides Enzymatiques PAP 1000, Bio-Merieux, Charbonnieres-les-Bains, France).

### *Body and tumour composition*

Body composition was determined on mouse carcasses stored at −20° after blood collection and removal of the tumour. The tumour was carefully freed of adhering muscle and weighed. The carcass or tumour were freeze-dried to constant weight, water content was calculated by difference. Total fat was determined by ethyl ether extraction of the desiccated material with a Soxhlet apparatus. After ash determination, protein was calculated by difference:

$$\text{Protein} = \text{Tissue weight} - (\text{water} + \text{ash} + \text{ether extract})$$

Body composition was determined for individual mice (ten mice per type of tumour and dietary regime), while tumour composition was determined in three pooled samples from each dietary treatment.

### *Statistical analysis*

Data were analysed by one-way analysis of variance. Differences between treatment means were assessed by Duncan's multiple range test (1955).

### Results

### *Food intake, water intake and body weight*

Food intake and body weight of the non-implanted mice serving as controls were affected significantly by the type of sugar. The F mice gained more weight and consumed more food as compared to the G mice whose body weight and food consumption were constant throughout the experimental period (Figure 1a and b). Water intake was highest at the start of the experiment, when the sugar enriched diets were offered, and decreased gradually thereafter (Figure 1c). In spite of the higher food intake, water consumption was lower in the F mice.

Tumour implantation was accompanied by a transient depression in food intake which was more pronounced in the F-fed mice than in the G group.

No consistent difference in water intake was observed between the tumour-bearing mice fed glucose and those receiving fructose, the water consumption in both groups being quite similar to that of the G control group.

The mean body weight of the tumour-bearing mice increased dramatically from day 10 after tumour implantation (Figure 2), while the body weights of the implanted mice in which no tumours were detected were similar to those of the non-implanted controls.

### *Tumour incidence and weight*

Tumours were detected in F fed mice earlier than in G fed ones (Figure 2). On day 15 after implantation, tumours were found in 23 mice of the F fed group as compared to 17 mice among the G fed animals. This difference subsisted till the end of the experimental period. Bigger tumours were observed in the F fed mice (Table II) which accounted for the higher body weight of this group.

### *Proximate composition of tumour (Table II) and carcass (Table III)*

There were no differences in tumour contents of water, fat and ash between the two dietary groups.

The water content was much higher in the thymoma-bearing mice than in the controls, fat concentration was

**Figure 1** Mean body weights, food intake and water intake per mouse of intact or tumour implanted mice receiving glucose (■—■) or fructose (+−−+) and of tumour implanted mice receiving glucose (*····*) or fructose (□−−□). Results for both dietary treatments up to the day of implantation were pooled. Vertical bars stand for the s.e.m.

lower by 40–50% and protein by 25%. The diet did not affect the body composition of the controls or the Thymoma-bearing mice.

### *Blood components (Table IV)*

Glucose and insulin levels were higher in the F fed control mice than in the G fed controls. Tumour implantation resulted in a lowered glucose level, while insulin levels increased dramatically. The response to tumour implantation was more pronounced in the F group than in G fed mice.

Triglycerides were higher in F group than in the G group, both in the implanted and control mice, but this diet effect was not statistically significant.

**Figure 2** Mean body weights of implanted mice which developed tumours. Figures shown on the upper two curves indicate the tumours detected in 30 mice. Tumour incidence and body weights of tumour-bearing mice were significantly higher for the fructose group than the the glucose-fed animals (paired $t$-test, $P<0.01$ and $P<0.05$ respectively). Vertical bars stand for the s.e.m. Glucose diet (+---+); fructose diet (■---■).

**Table II** Tumour weight[a] and composition[b] at autopsy

| Diet | Glucose | Fructose | S.E.M. |
|---|---|---|---|
| Tumour weight (g) | 1.33 | 2.26 | 0.12* |
| Water (%) | 7.05 | 6.89 | 0.38 NS |
| Fat (%) | 2.52 | 2.07 | 0.20 NS |
| Ash (%) | 1.58 | 1.70 | 0.08 NS |

[a]Mean values from 10-12 observations. [b]Mean values from three pooled samples from 3-4 mice each. *Statistically significant difference, $P<0.05$. NS indicates that the difference is not statistically significant.

**Table III** Carcass weight and composition of mice at autopsy

| | Control | | Thymoma-Implanted[1] | | |
|---|---|---|---|---|---|
| | Glucose | Fructose | Glucose | Fructose | S.E.M. |
| Weight (g) | 23.2[a] | 24.9[a] | 24.0[a] | 22.4[b] | 0.29 |
| Water (%) | 57.9[b] | 60.4[b] | 69.1[a] | 69.1[a] | 3.5 |
| Fat (%) | 10.0[a] | 9.11[a] | 6.12[b] | 4.95[b] | 0.76 |
| Ash (%) | 3.18[c] | 3.16[c] | 3.61[a] | 4.33[a] | 0.23 |
| Protein (%) | 27.3[a] | 28.9[a] | 21.6[b] | 21.4[b] | 0.95 |

Mean values from ten mice for control and tumour-implanted mice. Values within rows with different superscripts differ to a statistically significant degree, $P<0.05$. [1]Carcass without tumour.

### Discussion

Dietary fructose can produce changes in glucose and/or insulin metabolism (see Introduction). Therefore, it was of interest to verify its effects on mice implanted with an insulin-dependent tumour such as Thymoma, in comparison with animals receiving glucose.

Implanted Thymoma mice had low glucose and high insulin and triglyceride levels, which constitutes a characteristic feature of tumour-bearing animals, and was reported in our previous studies (Yam *et al.*, 1990*a*,*b*). It is of interest that the blood insulin level in tumour-bearing mice was especially high in the group receiving fructose. Dietary fructose also had hyperinsulinaemic effect in non-implanted control mice, more so than glucose, when compared to mice of the same strain receiving a sugar-free diet (Yam *et al.*, 1990*a*, *b*).

More importantly, dietary fructose enhanced the Thymoma growth, as seen from the earlier detection and larger size of the tumours. Tumour incidence appeared to be higher in the fructose group than in the glucose-fed animals (83% *vs* 57%), but this difference is of doubtful biological significance, because of the limited data on tumour take rate: previous experiments have shown that the take rate of the tumour in mice receiving the conventional pelleted diet is about 60%, i.e., similar to the glucose-fed animals.

The question therefore arises if and to what extent the increased tumour expression in the fructose fed mice may be related to insulin metabolism. There is some evidence that tumorigenesis in humans and laboratory animals is enhanced by elevated energy intake (Kritchevsky & Kleerfeld, 1986; Pariza, 1986; Graham, 1986; Kleerfield *et al.*, 1987), high consumption of mono and disaccharides (Risch *et al.*, 1985), starch (LaVecchia *et al.*, 1987), total fat and saturated fatty acids (Tonioli *et al.*, 1989) and omega-6 polyunsaturated fatty acids (Carroll, 1980; Hubbard & Erickson, 1987; Roebuck *et al.*, 1981).

All of these dietary factors are characterised by their enhancing effect on insulin secretion or by their interference with insulin metabolism (Hollenbeck & Reaven, 1987; Greenberger *et al.*, 1968; Montague & Taylor, 1968; Sanbar & Martin, 1967; Linscheer *et al.*, 1967; Lardinois *et al.*, 1987). Hyperinsulinaemia is sometimes accompanied by glucose intolerance (Hartog *et al.*, 1987) because of down-regulation of insulin receptors. This process is absent in tumour cells (Mountjoy *et al.*, 1987), and may be advantageous to these cells, especially in the case of insulin-dependent tumours.

It was reported by Enzmann *et al.* (1989), that feeding fructose caused a higher incidence of hepatocarcinoma in N-nitroso-morpholine-treated rats. This higher incidence was accompanied by an increase in hepatic glucose-6-phosphate and in the activity of glucose-6-phosphate dehydrogenase and by an excessive deposition of glycogen in the preneoplastic liver lesions. Unfortunately the insulin status was not ascertained in these rats. There too, this hormone may constitute a link between dietary fructose and tumour development.

It is concluded that high dietary fructose may enhance the incidence and growth of implanted or chemically induced murine tumours. The implications of the increasing fructose consumption referred to in the introduction deserve consideration in the context of human cancer.

**Table IV** Blood plasma or serum compositon

| | Control | | Thymoma-Implanted | | |
|---|---|---|---|---|---|
| Diet | Glucose | Fructose | Glucose | Fructose | S.E.M. |
| Glucose | | | | | |
| mg × (100 ml$^{-1}$) plasma | 145[b] | 170[a] | 83[d] | 58[c] | 3.6 |
| Insulin | | | | | |
| μU × ml$^{-1}$ serum | 3.2[d] | 35.3[c] | 56.3[b] | 78.0[a] | 2.3 |
| Triglycerides | | | | | |
| mg × (100 ml$^{-1}$) serum | 180[b] | 234[b] | 313[a] | 348[a] | 13.9 |

Measurements were carried out on three pooled samples of six mice each for each dietary treatment of control and tumour-implanted mice. Values within rows with different superscripts differ to a statistically significant degree, $P<0.05$.

### References

CARROLL, K.K. (1980). Lipids and carcinogenesis. *J. Environ. Pathol. Toxicol.*, **3**, 353.

DUNCAN, D.B. (1955). Multiple range and multiple F test. *Biometrics*, **11**, 1.

ENZMANN, H., OLHAUSER, D., DETTLER, T. & BANNASCH, P. (1989). Enhancement of hepatocarcinogensis in rats by dietary fructose. *Carcinogenesis*, **10**, 1247.

GRAHAM, S. (1986). Hypotheses regarding caloric intake in cancer development. *Cancer*, **58**, 1814.

GREENBERGER, N.J., TZAGOURNIS, M. & GRAVES, T.M. (1968). Stimulation of insulin secretion in man by medium chain triglycerides. *Met. Clin. Exper.*, **17**, 796.

HALLFRISCH, J., ELDWOOD, K.C., MICHAELIS, O.E., REISER, S., O'DORISIO, T.M. & PRATHER, E.S. (1983*a*). Effects of dietary fructose on plasma glucose and hormone responses in normal and hyperinsulinaemic men. *J. Nutr.*, **113**, 1819.

HALLFRISCH, J., REISER, S. & PRATHER, E.S. (1983*b*). Blood lipid distribution of hyperinsulinaemic men consuming three levels of fructose. *Am. J. Clin. Nutr.*, **37**, 740.

HARAN-GERA, N., BEN YAACOV, M. & PELED, H. (1977). Immunologic characteristics in relation to high and low leukomogenic activity of radiation of leukomogenic virus variants. *J. Immunol.*, **118**, 600.

HARTOG, J.M., LAMERS, M.J., MONTFORT, A. & 4 others (1987). Comparison of mackerel-oil and lard fat enriched diets on plasma lipids, cardiovascular performance and morphology in young pigs. *Am. J. Clin. Nutr.*, **46**, 258.

HOLLENBECK, C., & REAVEN, G.M. (1987). Variations in insulin-stimulated glucose uptake in healthy individuals with normal glucose tolerance. *Clin. Endocr. Metabol.*, **64**, 1169.

HUBBARD, N.E. & ERICKSON, K.I. (1987). Enhancement of metastasis from a transplantable mouse mammary tumour by dietary linoleic acid. *Cancer Res.*, **47**, 6171.

KLEERFELD, D.M., WEBER, M.W. & KRITCHEVSKY, D. (1987). Inhibition of chemically induced mammary and colon tumour promotion by caloric restriction in rats fed increased dietary fat. *Cancer Res.*, **47**, 2759.

KRITCHEVSKY, D. & KLEERFELD, D.M. (1986). Influence of caloric intake on experimental carcinogenesis. A review. *Adv. Exp. Med. Biol.*, **206**, 55.

LARDINOIS, C.K., STARICH, G.H., MAZZAFERI, E.L. & DE LETT, A. (1987). Polyunsaturated fatty acids augment insulin secretion. *J. Amer. College Nutr.*, **6**, 507.

LA VECCHIA, C., DECARLI, A., FRANCESCHI, S., GENTILE, A., NEGRI, E. & PARAZZINI, F. (1987). Dietary factors and the risk of breast cancer. *Nutr. Cancer*, **10**, 205.

LINSCHEER, W.G., SLONE, D. & CHAMBERS, T.C. (1967). Effects of octanoid on serum levels of free fatty acids, insulin and glucose in patients with cirrhosis and healthy volunteers. *Lancet*, **i**, 593.

MONTAGUE, W. & TAYLOR, K.W. (1968). Regulation of insulin secretion by short chain fatty acids. *Nature*, **217**, 853.

MOUNTJOY, K.G., FINLAY, G.J. & HOLDAWAY, I.M. (1987). Abnormal insulin receptor down regulation and dissociation of down regulation from insulin action in cultured human tumor cells. *Cancer Res.*, **47**, 6500.

PARIZA, M.W. (1986). Caloric restriction, ad libitum feeding and cancer. *Proc. Soc. Exp. Biol. Med.*, **183**, 293.

PAVELIC, K. & SLYJEPCEVIC, M. (1978). Growth of a Thymoma in diabetic mice treated with insulin. *Eur. J. Cancer*, **14**, 675.

PAVELIC, K., SLYJEPCEVIC, M., PAVELIC, J. & 4 others (1979). Growth and treatment of Ehrlich tumor in mice with alloxan induced diabetes. *Cancer Res.*, **39**, 1807.

PENNOCK, C.A., MURPHY, D., SELLERS, J. & LONGDON, K.J. (1973). A comparison of autoanalyser methods for the determination of glucose in blood. *Clin. Chim. Acta*, **48**, 193.

REISER, S. (1978). Effect of nutrient excess in animal and man: carbohydrates. In *CRC Handbook Series in Nutrition and Food, Section E: Nutritional Disorders*. Rechcigl, M. (ed.), pp. 409–436. CRC Press: West Palm Beach, FL.

REISER, S., POWELL, A.S., YANG, C.Y. & CANARY, J.J. (1987). An insulinogenic effect of oral fructose in humans during postprandial hyperglycemia. *Am. J. Clin. Nutr.*, **45**, 580.

RISCH, H.A., JAIN, M., CHAIB, K.J.P. & MILLER, A.B. (1985). Dietary factors and the incidence of cancer of the stomach. *Am. J. Epidemiol.*, **122**, 947.

ROEBUCK, B.D., YAGER, J.D. & LONGNECKER, D.S. (1981). Promotion by unsaturated fat of azarine induced pancreatic carcinogenesis in the rat. *Cancer Res.*, **41**, 3961.

SANBAR, S.S. & MARTIN, J.M. (1967). Stimulation of insulin release from isolated rat pancreas. *Met. Clin. Exp.*, **16**, 482.

SLEDER, J., CHEN, Y.D.I., CULLY, M.D. & REAVEN, G.M. (1980). Hyperinsulinemia in fructose-induced hypertriglyceridemia in the rat. *Metabolism*, **29**, 303.

THORBURN, A.W., STORLIEN, L.H., JENKINS, A.B., KHOURI, S. & KRAEGEN, E.W. (1989). Fructose-induced *in vivo* insulin resitance and elevated plasma triglycerides levels in rats. *Am. J. Clin. Nutr.*, **49**, 1153.

TONIOLO, P., RIBOLI, E., PROTTA, F., CHARREL, M. & CAPPA, A.P.M. (1989). Calorie-providing nutrients and risk of breast cancer. *JNCI*, **81**, 278.

YAM, D. (1988). Insulin involvement in malignancies. *J. Endocr. Invest.*, **11** (Suppl 1-4).

YAM, D., ZILBERSTEIN, A., FINK, A. & NIR, I. (1990*a*). Insulin-tumour interrelationships in EL-4 lymphoma or Thymoma-bearing mice I. Alloxan-diabetic or non-diabetic mice. *Br. J. Cancer*, **61**, 689.

YAM, D., FINK, A., NIR, I. & BUDOWSKI, P. (1990*b*). Insulin-tumour interrelationships in EL4-lymphoma or thymoma bearing mice II. Effects of dietary omega-3 and omega-6 polyunsaturated fatty acids. *Br. J. Cancer*, **62**, 897.

ZAVARONI, I., CHEN, Y.D.I. & REAVEN, G.M. (1982). Studies of the mechanism of fructose-induced hypertriglyceridemia in the rat. *Metabolism*, **31**, 1077.

ZAVARONI, I., SANDER, S., SCOTT, S. & REAVEN, G.M. (1980). Effect of fructose feeding on insulin secretion and insulin action in the rat. *Metabolism*, **29**, 970.